# Determination of factors that allow cryogenic nanoscopy with high power illumination without devitrification

**DOI:** 10.1371/journal.pone.0344631

**Published:** 2026-03-31

**Authors:** Jan Huebinger, Philippe I.H. Bastiaens

**Affiliations:** 1 Department of Systemic Cell Biology, Max Planck Institute of Molecular Physiology, Dortmund, Germany; 2 Faculty of Chemistry and Chemical Biology, TU Dortmund, Dortmund, Germany; Goa University, India, INDIA

## Abstract

Cryogenic fluorescence nanoscopy, also known as super-resolution fluorescence light microscopy, has been demonstrated to be useful to close the resolution gap in cryogenic correlative light and electron microscopy (CLEM). Importantly, under cryogenic conditions fundamental resolution barriers that are imposed by molecular motion and photobleaching on fluorescence microscopy are circumvented. Due to combination of intrinsically higher resolution on the one hand with strong photobleaching and slower acquisition speed on the other hand, nanoscopy methods show the greatest resolution gain under cryogenic conditions. Therefore, cryogenic fluorescence nanoscopy has great potential also beyond CLEM. However, cryogenic nanoscopy is often limited by heating of the sample through strong laser irradiation. If the sample is heated above the glass transition temperature, diffusion and conformational changes perturb the sample and the sample eventually devitrifies. Reported tolerable power densities on different systems vary by several orders of magnitude. We therefore investigated the laser-induced heating in different setups for cryogenic nanoscopy by time-dependent finite-element simulations complemented with absorption measurements of mammalian cells. This showed that laser-induced heating happens in milliseconds in these setups, precluding efficient sample preservation by intermittent illuminations unless the laser power is modulated in the kHz regime. Under moderate (kW/cm^2^) light densities used for single molecule localization microscopy, absorbance by mammalian cells was too weak to explain devitrification. Here, heating is governed by absorption of supporting material and can therefore be alleviated by optimizing this material. However, the much higher power densities used in stimulated emission depletion nanoscopy (MW/cm^2^) resulted in temperatures clearly above the devitrification temperature from absorption by cells alone. Therefore, samples should be mounted on an efficient heat exchanger, such as a diamond, for high power illumination schemes.

While cryo-fluorescence microscopy has been proven useful for cryogenic correlative light and electron microscopy/tomography (CLEM) [1–4], it has been also shown that cryogenic fluorescence microscopy breaks through a fundamental resolution barrier imposed by motional blur and photobleaching [5], rendering it also a very auspicious method for fluorescence micro- and nanoscopy outside of CLEM.

In order to cryo-arrest living cells in a ‘close-to-native’ state, they have to be cooled extremely rapidly below the glass transition temperature of water to avoid ice crystal formation in a process called vitrification [6,7]. Such ice crystals will exclude all solutes and cellular constituents and distort the sample before imaging [8,9]. The samples then have to be kept below the glass transition temperature during the whole preparation and imaging process to avoid devitrification. The absorption of energy from irradiation is an inevitable source of heating during fluorescence micro- or nanoscopy. This heating can be strong enough to lead to ice crystallization [3].

Upon heating above the glass transition temperature of water, an aqueous sample will first become fluid and then crystallize, if it remains sufficiently long between the glass transition and the melting temperature [10]. At temperatures close to the glass transition temperature, ice crystallization is relatively slow [11] and vitrification of aqueous solutions heated to this temperature regime can reoccur from a liquid state upon re-cooling [12]. Revitrification can also occur upon re-cooling, when a vitrified sample is heated above the melting temperature by laser irradiation and then cooled rapidly enough [11]. However, any heating of the sample above the glass transition temperature of water leads to a drastic increase in molecular motility and thus to changes in the sample, such as diffusion of molecules or conformational changes in proteins [13]. Since localization measurements with sub-nanometer precision and measurements of protein conformations are nowadays feasible by cryogenic fluorescence microscopy [5,14] and cryo-electron tomography [15], such changes should be avoided.

Single molecule localization microscopy (SMLM) [16] has been used to close the resolution gap between fluorescence light microscopy and electron microscopy (EM) in CLEM [3,4,17]. Since photobleaching has been shown to be dramatically reduced under cryogenic conditions [2,5,14], resolution in SMLM could be markedly improved under cryogenic conditions, despite the use of air objectives with relatively low numerical aperture (NA) [14,18,19]. However, it has also been noticed that prolonged exposure to laser light during typical SMLM illumination schemes (~300 Wcm^-2^) led to heating above the glass transition temperature of water, as evident from ice crystallization in the sample [3]. A combination of experiment and steady-state heat transfer simulations revealed that this heating was predominantly caused by absorption of the support film material and alternative support film material was identified that allowed higher illumination powers of approximately ~10^3^ Wcm^-2^ [20–22]. Other strategies to circumvent ice crystallization were reduction of illumination powers, intermittent irradiation profiles and/or addition of cryoprotective agents to the sample [3,23–26]. However, the use of cryoprotective agents is prone to change the state of living cells even before they are cryo-fixed [12,27] and reducing illumination powers limits the applicability of several nanoscopy methods. Further, the absence of ice crystals is not sufficient to prove that the sample is kept below the glass transition temperature of water. Changes to the sample, such as diffusion or protein conformational changes, can thus not be excluded by the absence of ice crystals.

More recently, stimulated emission depletion (STED) nanoscopy [28] has been used under cryogenic conditions [5]. These conditions enabled STED nanoscopy measurements in cells that were not possible at room temperature due to inhibition of motional blur and drastic reduction of photobleaching. STED nanoscopy has the advantage of being directly (semi-) quantitative and is thus of great interest for quantitative fluorescence microscopy. However, this advantage comes at the cost of much higher irradiation densities. Despite irradiation density (∼10^7^ Wcm^-2^) that were approximately 4 orders of magnitude higher than those used for cryo-SMLM on EM grids, no change in the sample was observed during continuous STED nanoscopy acquisition [5]. However, the state of the water was not directly measured, since no subsequent EM was performed.

One major difference in sample heating between the SMLM and STED nanoscopy measurements are the different sample mountings. For cryo-STED nanoscopy a thin (10−15 µm) aqueous sample containing living cells on a standard microscopy cover slide was mounted directly on a diamond heat exchanger of superior thermal conductivity (>10000 Wm^-1^K^-1^ at −196 °C) [29]. Cryo-SMLM was performed on an EM grid placed in vacuum or gaseous environment, where the heat had to be conducted laterally over relatively long distances through the thin film of aqueous sample of low thermal conductivity (<1 Wm^-1^K^-1^) or the support film material. On the other hand, the strongly focused (∼1.5 µm^2^) STED beam has a much greater surface to volume ratio than the widefield illumination used in SMLM (e.g., 100 µm^2^ in [3]), which can also contribute to faster heat dissipation. Additionally, illumination for SMLM is static and lasts for a relatively long time (typically several minutes), whereas the STED beam is scanned over the sample, so that each point in the sample is typically only directly illuminated for few to tens of milliseconds.

We therefore sought to unravel, which factors govern heating and devitrification upon laser irradiation. Additionally, we sought to investigate, if there is heating above the glass transition temperature in those conditions, where ice crystallization was not detectable or not tested. Lastly, we wanted to estimate the heating induced by STED nanoscopy on EM grids, to estimate if STED nanoscopy without heating above the glass transition temperature is feasible in this configuration.

To achieve this, we performed time-dependent finite-element simulations of laser-light absorption in the different sample configurations coupled to conductive heat transfer within those samples. To assess direct heating of mammalian cells by the different laser irradiations, we also determined the linear absorption coefficient of mammalian cells experimentally, since it was to our best knowledge hitherto unknown.

Time-dependent simulations showed that steady-state temperatures are generally reached in tens of millisecond after onset of irradiation. Thus, intermittent irradiation profiles still lead to heating above the glass transition temperature unless illumination is modulated in the kHz regime. We measured the absorption coefficient of mammalian cells to be 4 and 2 orders of magnitude higher than water at visible and near infrared irradiation wavelengths, respectively. Simulations of SMLM on EM grids showed that heating is nevertheless dominated by absorption of the support film and absorbance by mammalian cells alone is not sufficient for heating above the glass transition temperature. Our simulations confirm the reduced heating on holey gold or silver-coated carbon film [20–22]. Besides a higher thermal conductivity of these films, we found this to be largely based on high reflectivity of these films. Since reflection can be limiting for fluorescence microscopy and doesn’t allow to inspect sample parts outside of holes in the films. We therefore also simulated the heating of other film material and found SiO_2_ films as very promising low absorption material that would enable >100-fold stronger illumination. However, using the intense illumination of a scanning depletion beam for STED nanoscopy would heat cells on EM grids in an area of tens of micrometers around the scanning beam above the glass transition temperature even without any support film. This heating is drastically mitigated, when cells are mounted on a diamond heat exchanger, where temperatures stayed clearly below the glass transition temperature, despite this intense illumination.

## Results

### Heating of aqueous samples by SMLM and STED nanoscopy

**Fig 1:**
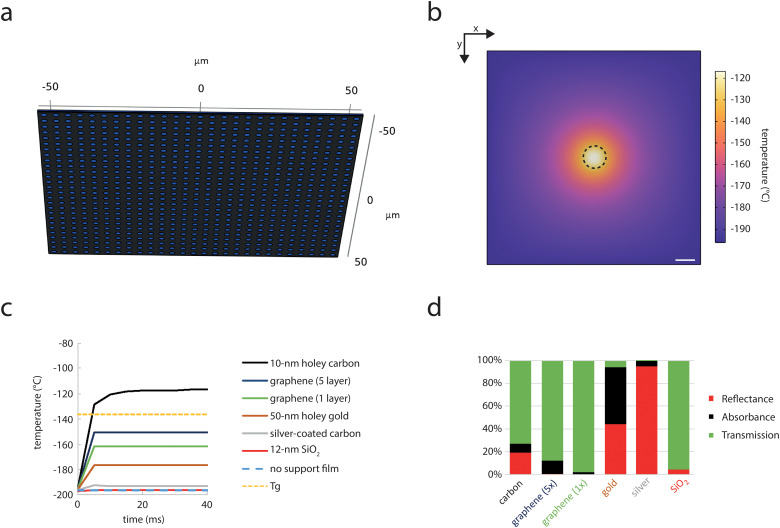
Heating of aqueous samples on EM grids by SMLM. a) The sample represents an individual square of an EM grid, where a 0.5 µm aqueous or cell layer (blue) was placed on a 10-nm carbon film (black) with holes of 2 µm diameters and interspacings of 2 µm. b) x-y view of the temperature profile of the EM grid close to steady-state after 40 ms of SMLM laser irradiation (0.315 mW) at a 100-µm^2^ spot in the center (black dashed circle). Scale bar: 10 µm c) Left: Temperature over time in the central point of the water layer above the central hole in the film from start of the SMLM laser irradiation in the EM grid configuration using a 10-nm holey amorphous carbon support (black line), a 50-nm holey gold support (brown line), a continuous 12-nm SiO_2_ support (red line), 1 or 5 layers of continuous graphene (dark blue and green lines), a 50-nm silver-coating on a holey amorphous carbon support (grey) or no support film (dashed blue line); Tg: glass transition temperature of water (dashed yellow line). d) Corresponding transmission (green), absorbance (black) and reflectance (red) of the support films at 488-nm and normal incidence.

We simulated first the heating in the central point of a 0.5-µm aqueous sample supported by a holey carbon film on an EM grid during SMLM illumination in a sample geometry as previously described [3,30]. Here, an EM grid is mounted to a brass block that is cooled by liquid nitrogen. To simulate optimal conditions, we assumed perfect conductivity between nitrogen, brass and grid that therefore represents a heat sink at −196°C. Thus, only the sample within the squares of the grid is heated by the laser beam (see method, Fig 1a). We simulated absorption of the laser-light in the different materials together with heat dissipation within these materials. To approximate an aqueous sample, we used published absorption values of water [31,32], the main constituent of buffers and cells. Upon laser irradiation with 0.3 mW at 488-nm plus 0.015 mW of 405-nm over an area of 100 µm^2^ (=300 + 15 Wcm^-2^), a new steady-state temperature between −108 °C and −115 °C, depending on beam shape, with an expected temperature gradient towards the periphery emerged. The steady-state temperature is approached by <1 °C difference within <20 ms (Fig 1b, 1c; S1 Fig; S1 Movie). This temperature is clearly above the glass transition temperature of water, where ice crystals will form, yet crystal growth is still relatively slow [11,12]. This result is therefore in very good agreement with the experimental observation that ice forms under such conditions, but only after minutes of exposure [3]. However, when the supporting carbon film was omitted in the simulation, temperature increased by less than 1 °C and thus stayed far below the glass transition temperature of water. We therefore also investigated the effects of alternative support films.

The higher transparency of formvar and silicon monoxide films had been shown to allow a higher irradiation density, but are also considered suboptimal for fluorescence microscopy and subsequent EM [21,23,25,26]. Commercially available holey 50-nm gold support films [33] and custom 50-nm silver-coating on holey carbon films [20] have been shown to heat less than holey carbon films [20–22]. We confirmed that both of the films heat less than holey carbon, with heating ratios that are very similar to previous reports (Fig 1c). While some of the benefits of these metal films comes from their higher thermal conductivity, it should be noted that the reduction in heating comes in large parts from the strong reflection of gold (~44%) and silver (>95%). The gold and silver films actually absorb >90% and >97% of the light that is not reflected on their surface, resulting in very low transmission of these films (Fig 1d; S2 Fig). This will mask information on parts of the sample that is not in the holes and strong reflections would impose strong requirements on the filtering of the detected light.

We therefore also tested silicon dioxide (SiO_2_) films [34] and continuous graphene films [35], which show much higher transmissivity (Fig 1d). Graphene has, despite its atomically thin layer (0.34 nm), a relatively high absorption of 2.3% per atomic layer [36], but can have very high in-plane thermal conductivity of >2000 Wm^-1^K^-1^ [37]. Graphene is typically used on top of a holey carbon film [35,38], but is also commercially available directly on EM grids. Simulations showed that despite a minimal reflectivity [39], aqueous samples on 1 or 5 layers of graphene were not heated above the glass transition temperatures. Here, it has to be however noted, that exact thermal conductivities of modified graphene layers that are typically used [33,38] are not exactly known (see Methods section). SiO_2_ support films can be around 12 nm thin [34] and have an orders of magnitude lower absorption coefficient (4.5x10^4^ m^-1^) and much lower reflection (~4%) at 488-nm compared to the other support film materials. A 12-nm amorphous SiO_2_ support film led to less than 1°C stronger heating compared to a sample without support film (Fig 1c) and laser intensities could be increased by a factor of 100 before the glass transition temperature of water was reached (S3 Fig).

**Fig 2:**
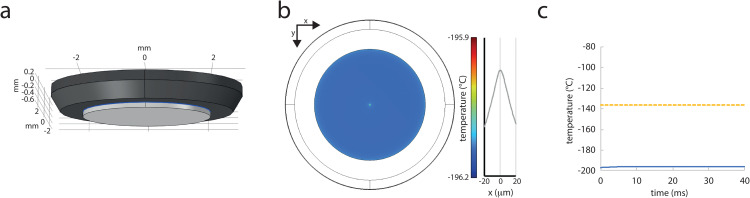
Heating of aqueous samples on a diamond heat exchanger by STED nanoscopy. a) The cryo-arrest sample was built as a 10-µm aqueous or cell layer (blue) between a 150-µm thick cover slide (light grey) and an 800-µm thick bevelled diamond disc (dark grey). b) x-y cross-sectional view of the temperature profile in the water layer close to steady-state after 40 ms of STED laser irradiation (1 W) at a 1.5-µm^2^ spot in the center. Right graph: Temperature distribution around the irradiated center. c) Temperature over time in the central point of the water layer from start of the STED laser irradiation (blue line); glass transition temperature of water is indicated by the dashed yellow line.

We next simulated the heating of a STED laser focused into an aqueous sample on a cover slide mounted to a diamond heat exchanger on the cryo-arrest stage, by simulating light absorption and heat dissipation in all three material layers. For this, the sample geometry was constructed as described [5](see methods, Fig 2a). Here, a constant irradiation with 1 W of 775-nm laser light at the central 1.5-µm^2^ spot (6.7x10^7^ Wcm^-2^) did not raise the temperature by more than 1 °C in this spot (Fig 2b, 2c).

### Measurement of absorption coefficient of mammalian cells

Cells absorb significantly more visible light than water, as evident from absorption measurements of cell suspensions [40,41], and will therefore be heated more strongly than water by laser irradiation. However, the simulation of heating of cells upon laser irradiation requires the linear absorption coefficient of the cells. This absorption coefficient cannot be extrapolated from suspension measurements, because the linear relationship between concentration and absorption is only valid for solutions and not suspensions [42–44].

**Fig 3:**
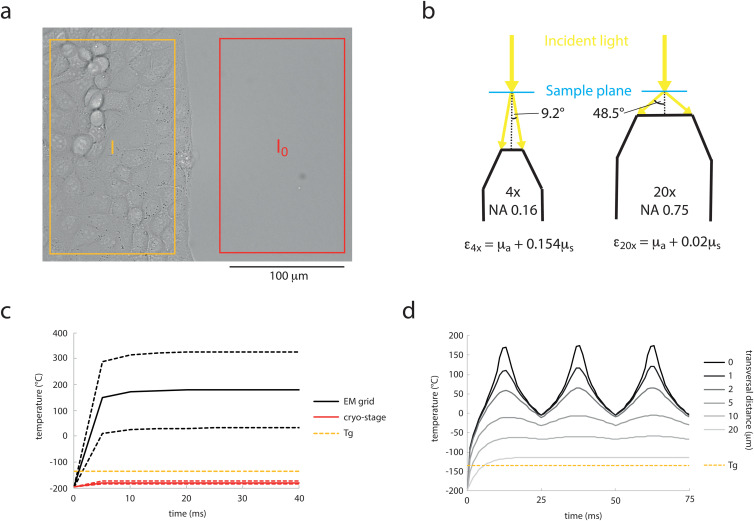
Absorption and heating of mammalian cells. a) Representative image of an extinction coefficient measurement using a 20x 0.75 NA objective at the border of a confined monolayer of MDCK cells. The transmitted light intensity after extinction by the cell monolayer (I) and the reference light intensity (I_0_) are measured in the same field of view. b) Schematic of the experiment for measuring the absorption coefficient of cells. Extinction coefficients (ε) were measured with a 4x 0.16 NA (left) and a 20x 0.75 NA (right) under non-convergent illumination. The extinction coefficient of the cells is composed of the absorption coefficient (μ_a_) and the fraction of the scattering coefficient (μ_s_) that misses the objective, i.e., that has a scattering angle larger than 9.2° (4x; 15.4±0.3%) or 48.5° (20x; 2±1%). c) Temperature over time in the central point of a cell layer (linear absorption coefficient 289±114m^-1^) from start of laser irradiation by a static 1-W STED laser focused to a 1.5-µm^2^ spot (~6.7x107 Wcm^-2^) in the EM grid configuration omitting a support film (black lines, solid: mean absorption coefficient, dashed: ±uncertainty) and on the ultra-rapid cryo-arrest stage (red lines); Tg: glass transition temperature of water (yellow dashed line) d) Evolution of temperature of a cellular sample (absorption coefficient: 289 m^-1^) on an EM grid during 3 lines of scanning (scanning range: 10-µm) with 40 nm transversal spacing by a 1-W STED laser. The temperature is depicted at the center of the 2nd scanning line (0) and at indicated transversal distances to the measurement point (gray lines). Tg: glass transition temperature of water (yellow dashed line).

To measure the mean absorption coefficient of mammalian cells experimentally, we cultured MDCK cells in confined monolayers. We first measured the extinction coefficient in a transmission light microscope (Fig 3a) together with measurements of cell heights by confocal laser scanning microscopy on the same samples (S4 Fig). Using a 20x 0.75NA objective, a coefficient of 639 ± 211 m^-1^ (mean ± sd; n = 6) was determined. However, this extinction coefficient contains not only absorption, but also the fraction of scattered light that was not collected by the objective. It can therefore only demark an upper limit to the absorption coefficient. Scattering of mammalian A375 cells and lymphocytes has been measured in dependence of the scattering angle [45] (S5 Fig). We therefore designed an experiment, where we used non-convergent illumination and measured the extinction coefficient with two different objectives with different NAs, i.e., two different collection angles. Using this approach and the published angle-dependent scattering, we were able to disentangle the scattering from the absorption coefficient (Fig 3b; Methods section), resulting in an absorption coefficient of 289 ± 114 m^-1^ (mean ± sem; N = 7; n = 68–162), which is 4 orders of magnitude higher than the absorption of water at 488 nm and 2 orders of magnitude higher than the absorption of water at 775 nm.

Using this absorption coefficient, we first simulated the heating of a cell by SMLM. When no support film was considered, this resulted in a heating of less than 2 °C (S6 Fig). This confirms that heating on an EM grid is practically only determined by the absorption of the support film, even if the absorption by the cells is considered, as long as strongly absorbing materials such as carbon or metal are used. However, for weakly absorbing support material, such as SiO_2_ films or cover slides, the direct heating of cells becomes relevant.

We then simulated heating by a static STED laser of a cell mounted to the diamond heat exchanger of the cryo-stage versus a cell on an EM grid without any support film. The latter would be a realistic scenario, if the scanning was confined to a hole in a holey support film. The temperature in the cellular sample on the diamond heat exchanger was clearly stronger elevated than the temperature in a water sample, but it also stayed clearly below the glass transition temperature of water. However, on an EM grid, temperatures were reached that would melt or even evaporate the sample locally (Fig 3c). When the sample thickness was varied between 0.5 and 5 µm, temperature increase varied by 2% with no particular trend (S7 Fig). This indicates that this reflects rather an uncertainty in the temperature prediction and the influence of the sample thickness is only minor. It should further be noted that above the melting temperature, convective heat transport becomes a significant factor. Therefore, the temperature development above 0°C cannot be reliably simulated by a conductive heat-transfer model. Further, in a real STED experiment, the laser is not static, but constantly scanning over the sample. We therefore also simulated scanning of the laser over a 10 µm wide field of view of the sample on an EM grid with a pixel length of 40 nm and a pixel dwell time of 100 µs (S2 Movie). Under these conditions temperatures approached the steady-state temperatures within a few °C when the scanning beam directly hits a region of the sample and the surrounding sample is constantly heated above the glass transition temperature within a distance >20 ± 10 µm (Fig 3d, S8 Fig).

## Discussion

To our knowledge, there is no previous report on the linear absorption coefficient of mammalian cells. We have therefore determined this value experimentally. Cellular absorption thereby tends to be higher than tissue absorption (3−160m^-1^) [46], which could be explained by the interstitial spaces absorbing less than the cells themselves. Also, a 30-40x higher scattering than absorption coefficient is consistent between cells and tissues. The relatively large uncertainty for the obtained cellular absorption originates in large parts from the large relative differences in angle dependent scattering (~±50% at 50°) between different cell types [45], which propagates as uncertainty into the absorption coefficient. The result therefore also reflects differences between cell types. Furthermore, it is to be expected that there are local absorption differences over individual cells due to sub-cellular spatial inhomogeneity. Depending on the cell type the samples absorption properties might also change, e.g., microorganisms that are usually much denser than mammalian cells or pigmented cells could be expected to absorb significantly more light. Despite these uncertainties, the results give clear indications under which conditions heating above the glass transition temperatures will occur.

In this study, we use the glass transition temperature of water as a stringent criterium. Replacing a significant amount of water by cryoprotectants would increase the glass transition temperature of the aqueous solution. E.g. replacing about 30% of the water by trehalose would increase the glass transition temperature to >-110°C [47], whereas salts have little impact on the glass transition temperature at concentrations usually used for cell culture buffers [48,49]. Using 20–30% of cryoprotectants is not unusual, especially for high-pressure frozen cells [50–52]. However, the accompanying water loss changes cells and should therefore optimally be avoided [12]. Both plunge-freezing and ultra-rapid cryo-arrest have been used without addition of cryoprotectants to the cells [3,5].

Chang et al. found ice formation on EM grids with carbon support films only after prolonged exposure time [3]. This is in very good agreement with the rapidly established steady-state temperature of ~-110°C. At such low temperatures, ice formation is slow [12]. It is therefore consistent that ice formation could be avoided by the use of cryoprotective agents and intermittent illumination schemes [3]. Also the dense procaryotic cytoplasm, that is much more cryoprotective then mammalian cytoplasm due to a lower water content [53], showed a cryoprotective effect in these borderline conditions [3]. The simulations are also in good agreement with other published results, where the threshold for long exposure times without ice formation in the absence of cryoprotective agents was found between 30 and 100 Wcm^-2^ [24–26], which would correspond to a laser power of 0.03–0.1mW in the tested setup. The absolute tolerable intensity will thereby also depend on excitation wavelength and profile (S1 Fig), illuminated area, size and interspacing of holes and grid mesh size. However, these factors appear to be relatively minor and all results are within the same order of magnitude.

Clearly, ice crystallization needs to be avoided in CLEM, since it is detrimental for EM. Ice-crystals are, however, also detrimental for fluorescence micro- or nanoscopy outside of CLEM, since water concentrates in ice-crystals and displaces all other molecules, leading to distortion of the sample [8,9]. Importantly, when samples are heated above the glass transition temperature of water without sufficient time to crystallize, diffusional and conformational changes in the sample are still occurring. This will also alter the sample in an uncontrolled way. Given that high-resolution data is nowadays obtainable by cryo-electron tomography, cryo-fluorescence nanoscopy or cryo-microspectroscopy [5,14,15], such changes can readily translate into distortions of the results. Our results show that temperature steady-states upon laser irradiation are approached within <10 ms. This shows that intermittent irradiation profiles will not prevent the sample from reaching temperatures above the glass transition unless they are modulated in the kHz regime. Lasers that are pulsed with tens of MHz are however too fast to allow sufficient cooling in between the pulses and will behave equivalent to continuously emitting light sources. Currently employed intermittent illumination protocols that illuminate for tens of seconds only prohibit ice crystallization by reducing the time segments above the glass transition. It is of note that the success of avoiding ice crystallization by intermittent illumination [3,23] therefore implies that it is not only integrated time above the glass transition temperature that governs ice crystallization, but the length of individual continuous time segments above the glass transition temperature appears to have a significant influence.

The presented results highlight the need of considering sample geometries for cryogenic fluorescence light microscopy. The use of highly absorbing support films should be avoided to prevent heating during fluorescence microscopy on EM grids [20–23]. However, the choice of support film material for CLEM is a multifactored problem, since support films are ideally very stable under the electron beam, ensure good sample adhesion, show low autofluorescence and high transmissivity [21,25,26,54]. Using the appropriate support film, allows a broad range of SMLM techniques to be used. In this context, it is of interest that a SiO_2_ support film allows a  > 100x higher illumination compared to standard holey carbon film, while maintaining a much higher transmissivity than silver or gold films.

However, for the use of strongly focused high intensity laser light, as used in STED nanoscopy, the additional mounting of the sample on an efficient heat exchanger appears necessary. Apart from the tested configuration where the sample is mounted to a heat exchanger made of diamond, which is cooled from the side opposing the sample directly by a flow of liquid nitrogen, also the mounting of a sample on a sapphire disc [4] can be considered. However, at the temperature of liquid nitrogen, diamond is the superior thermal conductor with thermal conductivities >10000 Wm^-1^K^-1^ [29] compared to sapphire (<1000 Wm^-1^K^-1^), which reaches its peak conductivity between 10 and 30 K [55] and can be a very efficient thermal conductor at even lower temperatures [4].

### Methods

#### Finite-element simulations.

Time-dependent finite-element simulations of absorption of a radiative beam and heat transfer in solid material were conducted in the COMSOL Multiphysics software Version 6.2 (COMSOL Inc., Burlington, MA). The attenuation of the laser beam was implemented via Beer-Lambert law


$$I=I_{\text{0}}e^{-\mu_{a}z}$$

Where I and $I_{\text{0}}$ are the attenuated and initial laser beam power densities, $\mu_{a}$ is the local absorption coefficient of the material that is penetrated by the beam and z is the penetration depth of the laser into the material.

In the finite-elements the equation solved is


$$\frac{\text{e}}{∥\text{e}∥}\nabla I=-\mu_{a}I$$

where e is the orientation of the beam.

The absorbed energy at each spatial element is then:


$$Q=\mu_{a}I$$

The absorbed energy represents a heat source, which is distributed within the sample via thermal conductivity in the solid materials:


$$Q=\rho C_{p}\frac{\partial T}{\partial t}+\nabla \cdot q$$

where ρ is the density, $C_{p}$ is the heat capacity at constant pressure and q=−κ∇T is the heat flux by conduction with κ being the thermal conductivity of the local material.

The geometries of the samples and their mountings were implemented as described in the original publications. Chang et al. used gold EM finder grids covered with a holey carbon film [56]. These were mounted into the cryostage^2^, a cryostage dedicated to CLEM on EM grids, where an EM grid is mounted to a brass block that is cooled by liquid nitrogen [1,30]. Since the thermal conductivity of brass (~110 Wm^-1^K^-1^) and gold (~310 Wm^-1^K^-1^) are orders of magnitude higher than those of water (<0.6 Wm^-1^K^-1^) or the supporting carbon film (0.5 Wm^-1^K^-1^; [57]), it was assumed that the brass and the gold adopted the temperature of the liquid nitrogen (−196°C) and are not significantly heated by the laser light. Therefore, one grid square with an edge length of 108 µm was modeled as a layer of water of 500 nm, as given by Chang et al., or up to 5 µm, which is the approximate height of a mammalian cell. The layer is supported by a carbon film with a thickness of 10 nm (Quantifoil Micro Tools GmbH, Großlöbichau, Germany) and 2-µm holes with 2 µm interspacing. One hole is placed exactly in the center of the film (Fig 1a). Alternatively, the sample was supported by a holey gold film [33] or a holey carbon film coated with 50 nm silver [20] of the same configuration or continuous films of 1 or 5 graphene layers [35] or a continuous 12-nm SiO_2_ film [34]. The temperature outside of the square was fixed to −196°C. Above and below the water and carbon are small cavities filled with air, which was modeled as complete insulation, because of the very low thermal conductivity of air (~0.01 Wm^-1^K^-1^) and because convective effects are largely blocked by the small cavities. Only very minor heat dissipation effects on EM grids have been observed when relatively large convective flows were considered [22]. Further, the temperature of the air cannot be taken as temperature of the coolant, because it is in contact with the warmer glass windows of the cryostage^2^. Thus, we consider here optimal cooling conditions, since the conduction from the liquid nitrogen over the brass block to the gold EM grid could lead to a small temperature gradient and conduction or convection by the air could lead to minor heating of the whole sample. The cryo-stage developed in [5] is dedicated to cryo-arrest during fluorescence live cell imaging and subsequent cryogenic fluorescence microscopy. Here, cells are grown on a standard microscopy cover slide and attached to a heat exchanger made out of chemical vapor deposited diamond that is cooled from the other side by a constant flow of liquid nitrogen. The diamond is mounted in a stainless-steel mount. Initial simulations showed that the stainless-steel mount has negligible effect on the temperature at the irradiated site. Therefore, this sample was modeled as a 10-µm aqueous layer on top of a 150-µm cover slide attached to a beveled diamond disk of 800 µm thickness and 3-mm radius (Fig 1b) and contact site to the stainless-steel were treated as insulation. The convective heat flux due to the 20 °C warm gas at the cover slide and the free parts of the diamond bottom were modeled with a constant heat flux of 5 Wm^-2^K^-1^, a standard value for indoor convection. However, as can be seen from Fig 2b, the temperature gradient from the sides is negligible and convection by surrounding gas has no significant influence on the temperature in the center of the sample. The geometries were modeled in 3D using a mesh to discretize the geometry into a finite number of tetrahedral elements. It was made sure to have a high enough number of finite elements in the area of the laser beam and that using a finer mesh did not significantly change the result of the simulation.

SMLM illumination was modeled as described by Chang et al., who illuminated with 0.3 mW of 488-nm laser light and 0.015 mW of 405-nm laser light. Since the absorption of water at 405-nm is approximately 2-fold higher compared to 488-nm laser light, an illumination intensity of 0.33 mW with the water absorption at 488-nm was used. The illumination area was given as 100 µm^2^ [3] and was modeled as a flat-top illumination profile, which is the preferred condition for SMLM [58]. However, we compared this profile also to a gaussian beam profile with a full width at half maximum of 5.6 µm (≈100 µm^2^). This led to only slightly elevated temperatures in the center of the beam (S1 Fig). STED illumination was modeled as a top-hat disk with a radius of 700 nm and a transition zone of 500 nm. The size closely resembles the measured doughnut shaped beam using a 0.95 NA air objective as used for cryo-STED [5] (S9 Fig). The area heated above the devitrification temperature is very large (>100 µm^2^) compared to the beam size (~1.5 µm^2^). Therefore, a more detailed description of the beam profile would not yield significant improvements in temperature distribution. The incident power was set to 1 W. This power is achievable in the sample, when commercially available 3-W lasers at 775-nm are used. The excitation laser power used for cryo-STED was less than 0.1% of the depletion laser power [5] and was therefore neglected.

Absorption coefficients of water (2.4 m^-1^ at a wavelength of 775 nm; 0.017 m^-1^ at 448 nm), thin film amorphous carbon (2.3x10^7^ m^-1^) and the glass cover slide (0.36m^-1^ at 775 nm) were taken from the literature [31,32,59–61]. Absorption of thin gold film is based on measured values for the real (ε_1_ = −2.24) and imaginary (ε_2_ = 3.98) part of the dielectric function at λ=490 nm for a 53-nm gold film [62]. From this the real (n) and imaginary (κ) part of the complex refractive index (ñ) was calculated:


$$n=\sqrt{(\sqrt{\left({\varepsilon_{\text{1}}}^{\text{2}}+{\varepsilon_{\text{2}}}^{\text{2}}\right)}+\varepsilon_{\text{1}})/\text{2}}$$

$$\kappa =\sqrt{(\sqrt{\left({\varepsilon_{\text{1}}}^{\text{2}}+{\varepsilon_{\text{2}}}^{\text{2}}\right)}-\varepsilon_{\text{1}})/\text{2}}$$

The absorption coefficients ($\mu_{\alpha }$) was then calculated as


$$\mu_{\alpha }=\frac{\text{4}\pi \kappa }{\lambda }$$

The values for n and κ for thin films of silver (n = 0.131 and κ = 2.8), SiO_2_ (n ≈1.5 and κ≈0.002) and amorphous carbon (n ≈ 2.5 and κ≈0.375) were taken from the literature [63–65]. This results in $\mu_{\alpha }=\text{7}.\text{2}*{\text{1}\text{0}}^{\text{7}}{\text{ m}}^{-\text{1}}$, $\mu_{\alpha }=\text{4}.\text{7}*{\text{1}\text{0}}^{\text{7}}{\text{m}}^{-\text{1}}$ and $\mu_{\alpha }=\text{4}.\text{5}*{\text{1}\text{0}}^{\text{4}}{\text{m}}^{-\text{1}}$at λ=488 nm for silver, gold and SiO2, respectively. Reflections were calculated using Fresnels equation for normal incidence:


$$R={\left|\frac{{\tilde{\text{n}}}_{\text{1}}-{\tilde{\text{n}}}_{\text{2}}}{{\tilde{\text{n}}}_{\text{1}}+{\tilde{\text{n}}}_{\text{2}}}\right|}^{\text{2}}=\frac{{{(n}_{\text{1}}-n_{\text{2}})}^{\text{2}}+{{(\kappa }_{\text{1}}-\kappa_{\text{2}})}^{\text{2}}}{{{(n}_{\text{1}}+n_{\text{2}})}^{\text{2}}+{{(\kappa }_{\text{1}}+\kappa_{\text{2}})}^{\text{2}}}$$

where n_1_=1 and $\kappa_{\text{1}}$=0 refer to the values of air.

Angle-dependent reflections were also calculated as the average between parallel ${(R}_{∥})$ and perpendicular $\left(R_{⊥}\right)$polarized light:


$$R_{∥}={\left|\frac{{\tilde{\text{n}}}_{\text{2}}\text{cos}\left(\alpha_{\text{1}}\right)-{\tilde{\text{n}}}_{\text{1}}\text{cos}\left(\alpha_{\text{2}}\right)}{{\tilde{\text{n}}}_{\text{2}}\text{cos}\left(\alpha_{\text{1}}\right)+{\tilde{\text{n}}}_{\text{1}}\text{cos}\left(\alpha_{\text{2}}\right)}\right|}^{\text{2}}$$

$$R_{⊥}={\left|\frac{{\tilde{\text{n}}}_{\text{1}}\text{cos}\left(\alpha_{\text{1}}\right)-{\tilde{\text{n}}}_{\text{2}}\text{cos}\left(\alpha_{\text{2}}\right)}{{\tilde{\text{n}}}_{\text{1}}\text{cos}\left(\alpha_{\text{1}}\right)+{\tilde{\text{n}}}_{\text{2}}\text{cos}\left(\alpha_{\text{2}}\right)}\right|}^{\text{2}}$$

where ñ_1_ = 1 refers to the value of air, $\alpha_{\text{1}}$ is the angle of the incidence beam to the normal and $\alpha_{\text{2}}$ the angle of the refracted beam to the normal. The maximal illumination angle depends on the NA of the objective. However, in the range of illumination angles covered by air objectives, changes in reflectance are relatively minor (S2 Fig). Reflection is incorporated by attenuating the incident laser beam by the reflected fraction.

The transmission (T) of the support material was calculated as:


T=1−(A+R)

With the absorption (A) in a film of a given thickness (z):


$$A=e^{-\mu_{a}z}$$

The reported 2.3% of absorption in an atomic layer of graphene [36] were directly implemented as a heat source of 100 µm^2^ with 7.59 Wcm^-2^ in the thermally thin material. Thermal conductivity of the graphene was modelled as asymmetric with 2000 Wm^-1^K^-1^ in plane and 20 Wm^-1^K^-1^ perpendicular to the graphene layer. Free graphene layers can have higher in-plane conductivities [37]. However, the conductivity is reduced when the layer is in contact with other material and the graphene layers used on EM grids are usually modified graphene layers [38] and their exact thermal conductivities were unknown. The absorption coefficient of the chemical vapor deposited diamond at 775-nm (14 m^-1^) was given by the manufacturer (Element Six, London, UK). We used thermal conductivities of thin films of gold (72 Wm^-1^K^-1^) and silver (64 Wm^-1^K^-1^) as calculated by [20], based on formulas provided by [66], and also used by [22]. Cells have been shown to have variable thermal conductivities that are however in the same order as water, therefore we have used the same thermal conductivities for water (~0.6 Wm^-1^K^-1^ at 20°C) and cells [67,68].

#### Measurement of STED beam size.

A sample of 150-nm gold beads embedded in DPX mounting medium (Abberior Instruments GmbH, Göttingen, Germany) was scanned with 20 nm pixel length on a commercial confocal laser-scanning STED microscope (Expert Line; Abberior Instruments GmbH, Göttingen, Germany) equipped with a 775 nm wavelength STED laser (1.25 W), a vortex phase plate to create the doughnut-shaped laser beam profile, a 40x 0.95NA objective (UPlanApo; Olympus Deutschland GmbH, Hamburg, Germany) and a photomultiplier tube.

#### Cell culture.

MDCK cells (ATCC No. CCL-34) were obtained from ATCC. The cells were regularly tested for mycoplasma infection using the MycoAlert Mycoplasma detection kit (Lonza, Basel, Switzerland). They were maintained in Dulbecco’s Modified Eagle’s Medium (DMEM) supplemented with 10% fetal bovine serum (FBS), 200 mM L-Glutamine, and 1% nonessential amino acids and cultured at 37 °C with 95% air and 5% CO_2_.

#### Determination of absorption coefficient of mammalian cells.

MDCK cells were seeded in 2-well silicon culture inserts with a 0.22 cm^2^ area per well (IBIDI GmbH, Gräfelfing, Germany) that were placed into 2-well cell culture chambers on glass cover slides (Sarstedt AG & Co. KG, Nürnbrecht, Germany), as described before [69,70]. 0.25x10^4^ - 1x10^4^ cells were seeded per silicon well, forming a confined monolayer of cells with a clear border after removal of the silicon insert 1–2 days after seeding. The cell culture medium was replaced by 1 mL cell culture medium without phenol red, to have a more transparent medium, and without FCS, to minimize growth factor-induced cell migration [69,70].

Transmission images of the border of the monolayer were acquired using an Olympus IX 81 microscope equipped with a 4x 0.16 NA and a 20x 0.7 NA objective and an Orca ER camera. This way the intensity with (I) and without ($I_{\text{0}}$) extinction by the cell monolayer could be measured in the same image (Fig 3a). By this, $I_{\text{0}}$ readily corrects for all intensity losses such as reflections or absorption by the cell culture medium. The condenser of the microscope was removed to avoid convergence of the illumination. This way the NA of the objective reflects the collection angle of light (α) as NA=sin(α) for these air objectives (Fig 3b). Additionally, wells containing only cell culture medium without phenol red were imaged in order to correct for inhomogeneous illumination.

Afterwards, 10 μL of fluorescein solution was added to each sample and confocal stacks were recorded at a LeicaSP8 microscope equipped with a 60x 1.4 NA oil immersion objective using a white light laser for 488-nm excitation (S5 Fig). From these measurements the height of the monolayer in these samples was measured to be z=5.8±0.3μm (mean ± sem).

The extinction coefficients (ε) were calculated using:


$$\begin{array}{c} I=I_{\text{0}}\;*e^{-\varepsilon z} \end{array}$$

Thus:


$$\varepsilon =-\text{ln}(\frac{I}{I_{\text{0}}})/z$$

This resulted in $\varepsilon_{\text{2}\text{0}x}=\text{5}\text{0}\text{5}\pm \text{2}\text{9}{\text{m}}^{-\text{1}}$ and $\varepsilon_{\text{4}x}=\text{1}\text{9}\text{5}\text{3}\pm \text{1}\text{4}\text{0}{\text{m}}^{-\text{1}}$.

The extinction coefficient is a sum of the absorption coefficient ($\mu_{a})$ and a fraction (x) of the scattering coefficient ($\mu_{s})$ that reflects the light that is deflected strong enough to miss the detector, i.e., miss the objective:


$$\varepsilon =\mu_{a}+\text{x}\mu_{s}$$

84.6±0.3% and 98±1% of the light scattered by a cell are collected by 4x 0.16NA (α=9.2°) and 20x 0.75NA (α=48.5) objective [45] (S6 Fig). This results in a solvable set of equations:


$$\varepsilon_{\text{2}\text{0}x}=\mu_{a}+\text{0}.\text{0}\text{2}\mu_{s}$$

$$\varepsilon_{\text{4}x}=\mu_{a}+\text{0}.\text{1}\text{5}\text{4}\mu_{s}$$

Solving the system of equations:


$$\mu_{s}=(\varepsilon_{\text{2}\text{0}x}-\varepsilon_{\text{4}x})/-\text{0}.\text{1}\text{3}\text{4}$$

$\mu_{a}=\varepsilon_{\text{4}x}-\text{0}.\text{1}\text{5}\text{4}\mu_{s}$ or $\mu_{a}=\varepsilon_{\text{2}\text{0}x}-\text{0}.\text{0}\text{2}\mu_{s}$

Including standard error propagation, this resulted in $\mu_{s}=\text{1}\text{0}\text{8}\text{0}\text{6}\pm \text{1}\text{2}\text{3}\text{6}m^{-\text{1}}$ and $\mu_{a}=\text{2}\text{8}\text{9}\pm \text{1}\text{1}\text{4}m^{-\text{1}}$.

## Supporting information

S1 Fig Comparison of flat-top and gaussian beam profile. Temperature over time in the central point of the water layer from start of the 488-nm laser irradiation (0.33 mW) at a spot in the center in the complete EM grid configuration using a 10-nm carbon support using a flat-top irradiation profile of 5.6 µm radius (100-µm^2^; black line) versus a gaussian beam profile of 5.6 µm radius at half maximum (gray lines); Tg: glass transition temperature of water (dashed yellow line).
(PDF)

S2 Fig Reflectance as a function of maximal illumination angle. Shown is reflectance of the indicated support film material calculated using Fresnel equations. Reflectance of parallel and perpendicular polarized fractions were averaged and reflections are shown as the mean over the range of collection angles to the respective maximum.
(PDF)

S3 Fig Heating of water sample by SMLM on a SiO_2_ support. Temperature over time in the central point of the water layer from start of the 488-nm laser irradiation at a 100-µm^2^ (3.3x10^2^ – 3.3x10^5^ W/cm^2^) spot in the center in the complete EM grid configuration using a 12-nm SiO_2_ support and different laser intensities (gray lines); Tg: glass transition temperature of water (dashed yellow line).
(PDF)

S4 Fig Cell thickness measurement by confocal laser scanning microscopy. Representative image of a thickness measurement of a monolayer of MDCK cells. After addition of fluorescein solution, confocal stacks of the edge of confined monolayers of MDCK cells used for extinction measurements (figure 3a) were recorded. Shown is a reconstruction of an xz-plane that was used to measure the thickness of the cell layer along the z-axis. Scale bar: 10 μm.
(PDF)

S5 Fig Angle-dependent scattering of mammalian cells. Scattering intensities over scattering angle were extracted from Watson et al., 2004, Biophysical Journal (Figures 6 and 12). The missing last 20° (A375) or 30° (lymphocyte) values were extrapolated from the previous 30° using exponential functions with R^2^ = 0.999 and 0.97, respectively. Areas under the curves were calculated every 10°.
(PDF)

S6 FigHeating of a cellular sample by SMLM on an EM grid.Temperature over time in the central point of the cell layer (absorption coefficient 289 ± 114m^-1^) from start of the 488-nm laser irradiation at a 100-µm^2^ spot in the center of the complete EM grid configuration omitting the support film (black lines; mean ± sem); Tg: glass transition temperature of water (dashed yellow line).(PDF)

S7 FigHeating dependence on sample thickness.Temperature increase (ΔT) of cellular samples (absorption coefficient 289 m^-1^) upon irradiation by a static 1-W STED laser (~1.5 µm^2^) on an EM grid without considering the support film for various indicated sample thicknesses between 0.5 and 5 µm.(PDF)

S8 Fig Temperature course during scanning of cells on an EM grid. Temperature course during scanning of 3 10-µm lines by the 1-W STED laser at −40 nm, 0 nm and +40 nm in transversal direction relative to the measurement point over a cellular sample with the minimum determined absorption coefficient of 175 m^-1^ and the maximum determined absorption coefficient of 403 m^-1^ (b). The temperature is depicted at the center of the 2^nd^ scanning line (0) and at indicated transversal distances to the measurement point (gray lines). Tg: glass transition temperature of water (yellow dashed line).
(PDF)

S9 Fig STED laser beam size. a) The doughnut-shaped illumination profile of a was measured by reflection from a 150-nm gold particle using a 40x 0.95 NA objective. Scale bar: 1 µm b) A background-corrected line profile through the doughnut-shape in a) is compared to the top-hat shape that is used for the simulation. Both profiles have been normalized to an area under the curve of 1.
(PDF)

S1 Movie Heating of an aqueous sample on an EM grid. Shown is the temperature following irradiation of a circular region of 100 μm^2^ in the center of the gold EM grid mesh with a holey carbon support film by 0.33 mW of 488-nm light over 40 ms as simulated by finite-element simulation. Temperature is color-coded as indicated on the right.
(AVI)

S2 Movie Heating of a cell sample on an EM grid by a scanning STED laser. Shown is the irradiation intensity (left, color-code in W/m^2^) and the temperature (right, color-code in °C) following scanning of a 1-W STED laser focused to a radius of 0.7 μm over three 10-μm lines with a scanning speed to acquire 40-nm pixel length with a pixel dwell time of 100 μs as simulated by finite-element simulation. The temperature was simulated by absorption of the laser light by the cells with an absorption coefficient of 289 m^-1^ without considering absorption by a support film. The lines were scanned in the center of a gold EM grid mesh (see figure1).
(AVI)

S1 Data Data file corresponding to Fig 1. (XLSX)

S2 Data Data file corresponding to Fig 2. (XLSX)

S3 Data Data file corresponding to Fig 3 and Supplement. (XLSX)
