# Supplementary figures and images for: Determination of factors that allow cryogenic nanoscopy with high power illumination without devitrification
Source: PLoS One. 2026 Mar 31;21(3):e0344631. doi: 10.1371/journal.pone.0344631 (PMC13038024; doi:10.1371/journal.pone.0344631)

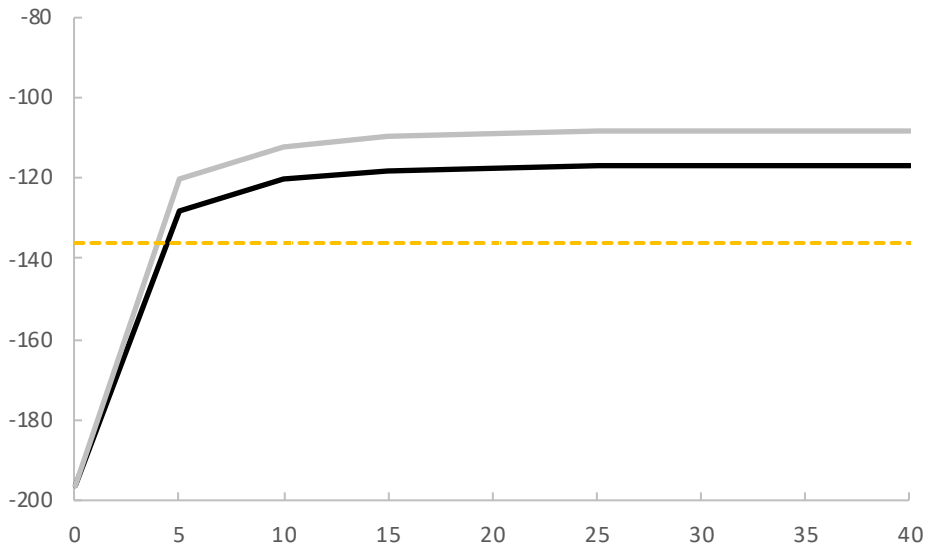

Supplement: S1 Fig — Temperature over time in the central point of the water layer from start of the 488-nm laser irradiation (0.33 mW) at a spot in the center in the complete EM grid configuration using a 10-nm carbon support using a flat-top irradiation profile of 5.6 µm radius (100-µm2; black line) versus a gaussian beam profile of 5.6 µm radius at half maximum (gray lines); Tg: glass transition temperature of water (dashed yellow line). (PDF) [file pone.0344631.s001.pdf]

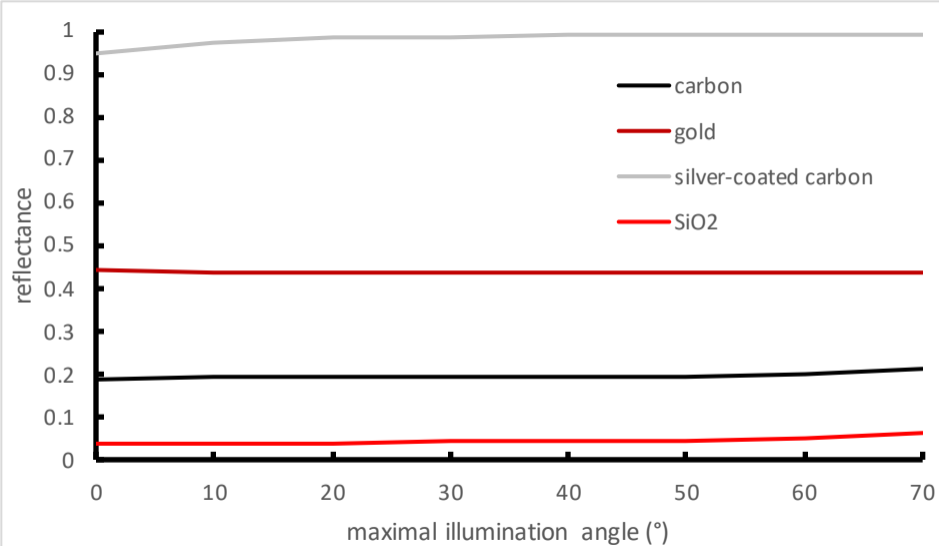

Supplement: S2 Fig — Shown is reflectance of the indicated support film material calculated using Fresnel equations. Reflectance of parallel and perpendicular polarized fractions were averaged and reflections are shown as the mean over the range of collection angles to the respective maximum. (PDF) [file pone.0344631.s002.pdf]

temperature (°C)

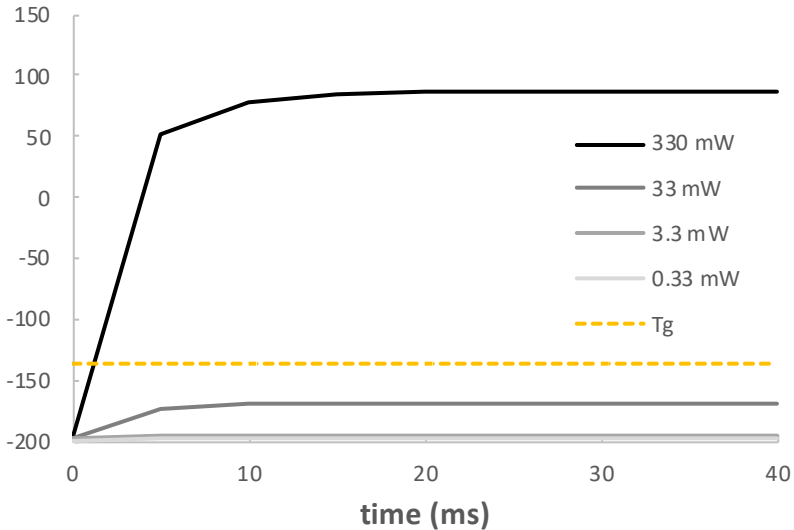

Supplement: S3 Fig — Temperature over time in the central point of the water layer from start of the 488-nm laser irradiation at a 100-µm2 (3.3x10^2^ – 3.3x10^5^ W/cm2) spot in the center in the complete EM grid configuration using a 12-nm SiO2 support and different laser intensities (gray lines); Tg: glass transition temperature of water (dashed yellow line). (PDF) [file pone.0344631.s003.pdf]

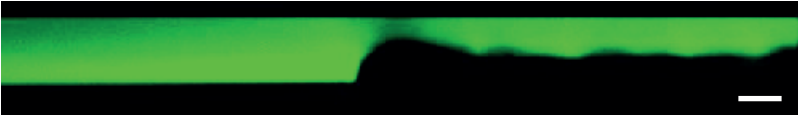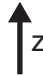

Supplement: S4 Fig — Representative image of a thickness measurement of a monolayer of MDCK cells. After addition of fluorescein solution, confocal stacks of the edge of confined monolayers of MDCK cells used for extinction measurements (figure 3a) were recorded. Shown is a reconstruction of an xz-plane that was used to measure the thickness of the cell layer along the z-axis. Scale bar: 10 μm. (PDF) [file pone.0344631.s004.pdf]

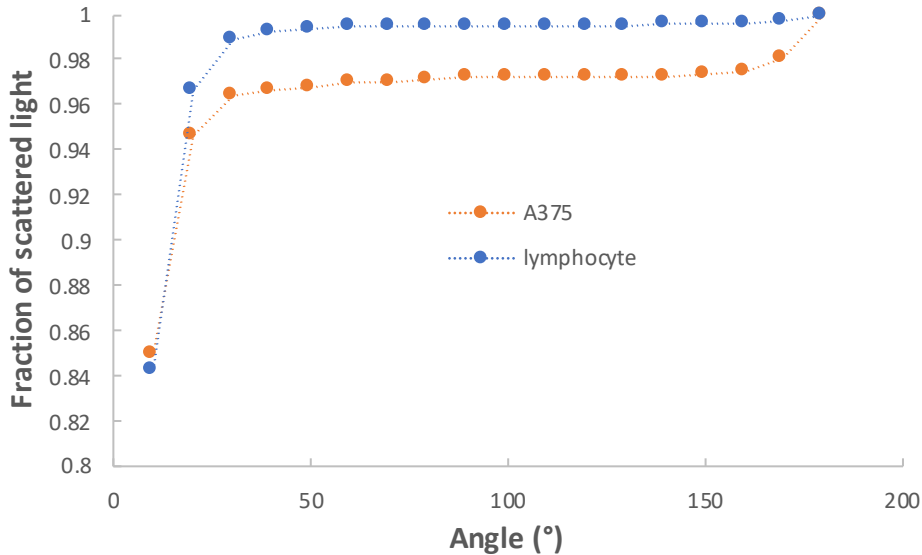

Supplement: S5 Fig — Scattering intensities over scattering angle were extracted from Watson et al., 2004, Biophysical Journal (Figures 6 and 12). The missing last 20° (A375) or 30° (lymphocyte) values were extrapolated from the previous 30° using exponential functions with R2 = 0.999 and 0.97, respectively. Areas under the curves were calculated every 10°. (PDF) [file pone.0344631.s005.pdf]

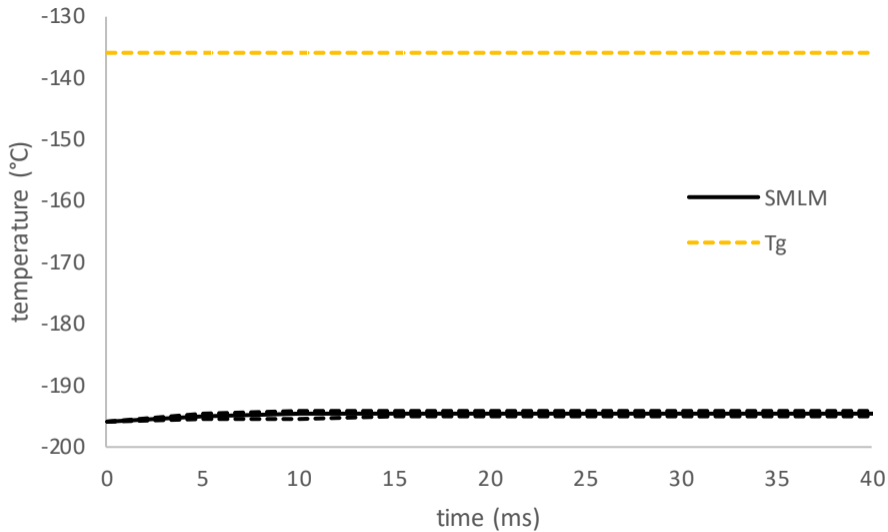

Supplement: S6 Fig — Temperature over time in the central point of the cell layer (absorption coefficient 289 ± 114m-1) from start of the 488-nm laser irradiation at a 100-µm2 spot in the center of the complete EM grid configuration omitting the support film (black lines; mean ± sem); Tg: glass transition temperature of water (dashed yellow line). (PDF) [file pone.0344631.s006.pdf]

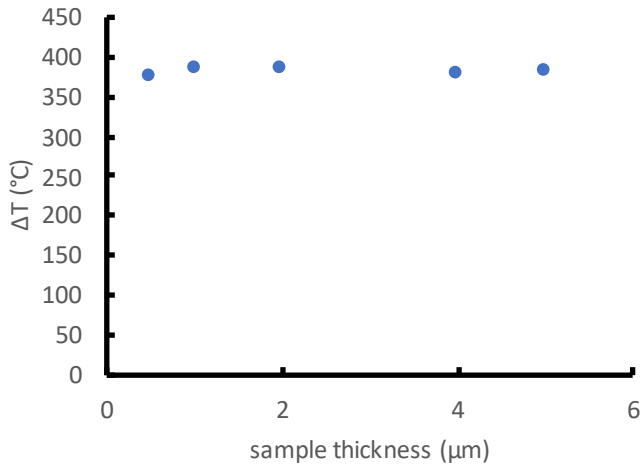

Supplement: S7 Fig — Temperature increase (ΔT) of cellular samples (absorption coefficient 289 m-1) upon irradiation by a static 1-W STED laser (~1.5 µm2) on an EM grid without considering the support film for various indicated sample thicknesses between 0.5 and 5 µm. (PDF) [file pone.0344631.s007.pdf]

a

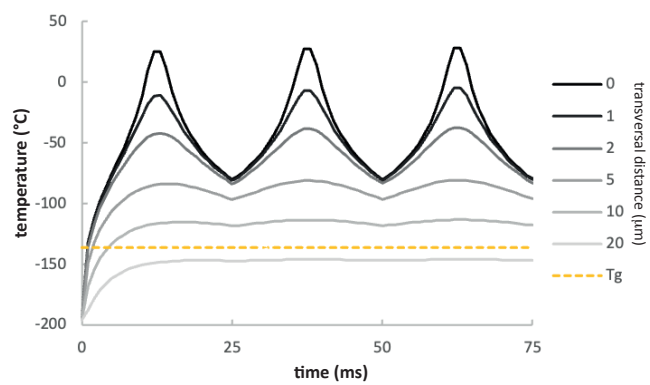

b

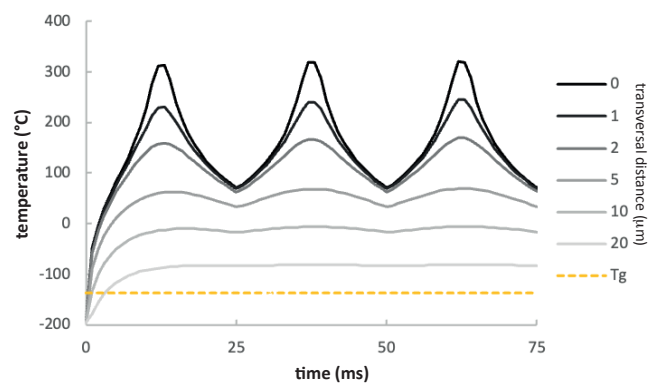

Supplement: S8 Fig — Temperature course during scanning of 3 10-µm lines by the 1-W STED laser at −40 nm, 0 nm and +40 nm in transversal direction relative to the measurement point over a cellular sample with the minimum determined absorption coefficient of 175 m-1 and the maximum determined absorption coefficient of 403 m-1 (b). The temperature is depicted at the center of the 2nd scanning line (0) and at indicated transversal distances to the measurement point (gray lines). Tg: glass transition temperature of water (yellow dashed line). (PDF) [file pone.0344631.s008.pdf]

a

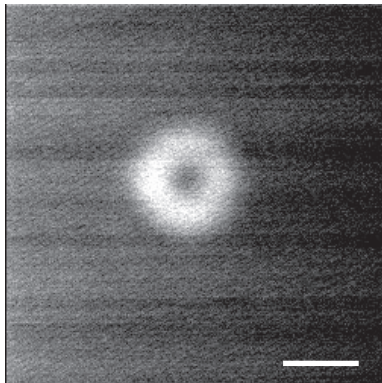

b

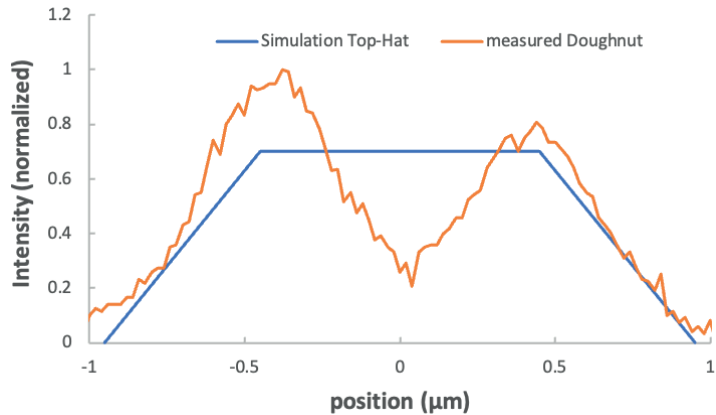

Supplement: S9 Fig — a) The doughnut-shaped illumination profile of a was measured by reflection from a 150-nm gold particle using a 40x 0.95 NA objective. Scale bar: 1 µm b) A background-corrected line profile through the doughnut-shape in a) is compared to the top-hat shape that is used for the simulation. Both profiles have been normalized to an area under the curve of 1. (PDF) [file pone.0344631.s009.pdf]
